# Fortified Settlements of the 9th and 10th Centuries ad in Central Europe: Structure, Function and Symbolism

**DOI:** 10.1179/0076609712Z.0000000003

**Published:** 2012-11

**Authors:** Hajnalka Herold

**Affiliations:** 1 VIAS — Vienna Institute of Archaeological Science, University of Vienna, Franz-Klein-Gasse 1, A-1190 Vienna, Austria. *hajnalka.herold@univie.ac.at*

## Abstract

THE STRUCTURE, FUNCTION(S) and symbolism of early medieval (9th–10th centuries ad) fortified settlements from central Europe, in particular today’s Austria, Hungary, Czech Republic and Slovakia, are examined in this paper. It offers an overview of the current state of research together with new insights based on analysis of the site of Gars-Thunau in Lower Austria. Special emphasis is given to the position of the fortified sites in the landscape, to the elements of the built environment and their spatial organisation, as well as to graves within the fortified area. The region under study was situated on the SE border of the Carolingian (and later the Ottonian) Empire, with some of the discussed sites lying in the territory of the ‘Great Moravian Empire’ in the 9th and 10th centuries. These sites can therefore provide important comparative data for researchers working in other parts of the Carolingian Empire and neighbouring regions.

Fortified settlements were important centres in the later phases of the central European early Middle Ages (9th–10th centuries ad). Their study therefore constitutes an essential part of early medieval archaeology in this region. This paper examines fortifications located in contemporary Austria, Hungary, Czech Republic and Slovakia. (See Fig 1 for the location of sites mentioned in the text.) It should be noted that research traditions have played a significant role in the relative emphasis placed on different aspects of early medieval archaeology in the regions concerned. Research in Austria and Hungary has mainly concentrated on cemeteries, while the excavation of fortified settlements has been a major concern of early medieval archaeology in the Czech Republic and Slovakia (united as Czechoslovakia until 1992).

**Figure 1:**
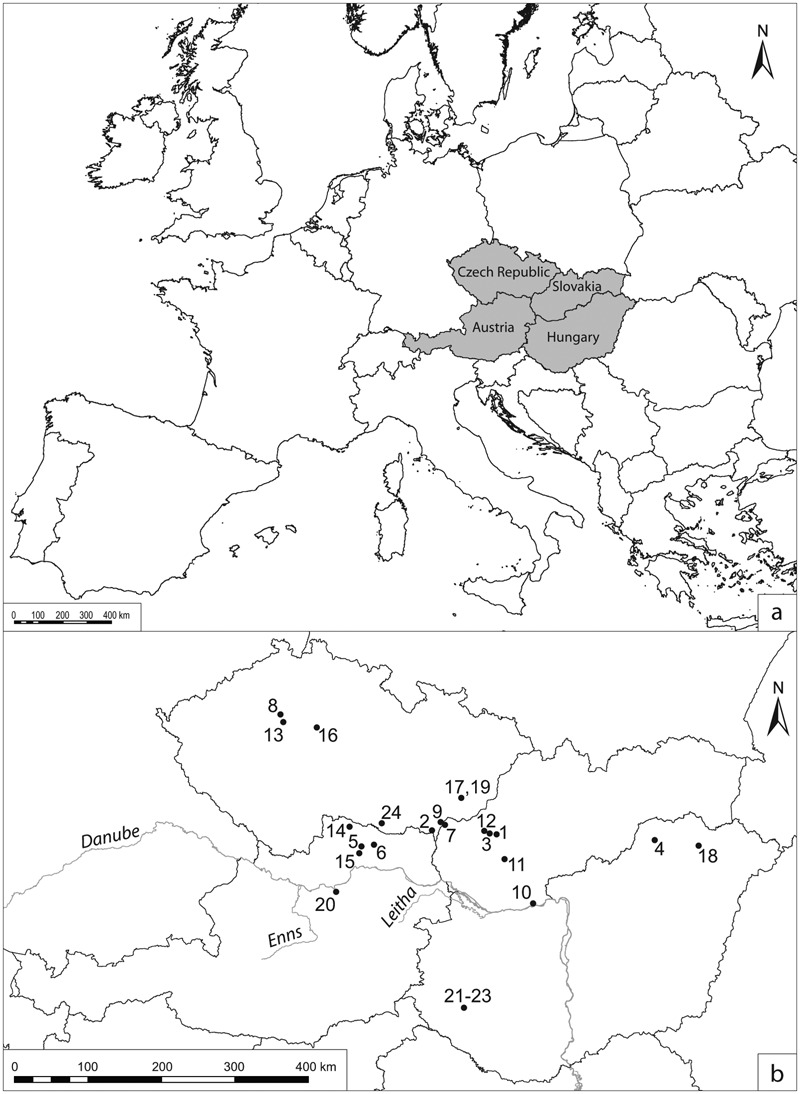
(a) Location map of the study area (grey) in Europe. (b) Location map of the sites and rivers mentioned in the text. Sites: **1** Bojná. **2** Břeclav-Pohansko. **3** Ducové. **4** Edelény-Borsodi földvár. **5** Gars-Thunau. **6** Heidenstatt near Limberg. **7** Kopčany. **8** Levý Hradec. **9** Mikulčice. **10** Mužla-Čenkov. **11** Nitra. **12** Pobedim. **13** Praha/Prague. **14** Sand near Oberpfaffendorf. **15** Schiltern-Burgstall. **16** Stará Kouřim. **17** Staré Město. **18** Szabolcs. **19** Uherské Hradiště-Sady. **20** Wieselburg. **21** Zalaszabar-Borjúállás sziget. **22** Zalavár-Récéskút. **23** Zalavár-Vársziget. **24** Znojmo-Hradiště. Rivers: Danube, Enns and Leitha.

The fortified settlements examined here have different origins; some were founded in the early 9th or possibly late 8th centuries ad, with others being established in the later 9th and 10th centuries. Although settlement remains and/or graves from the 7th and early/mid-8th centuries are present at some of these sites, settlement activity became more intensive during the 9th and 10th centuries in these places. The intensification of settlement activity and the emergence of fortified sites indicate basic changes in the political, economic and social history of the area concerned around the beginning of the 9th century, as well as multiple transformations during the following two centuries. This article provides a short overview of the historical background, and then examines different aspects of the archaeology of these fortified sites.^2^

## HISTORICAL BACKROUND

In the 8th century the major polities in this region of central Europe were the Duchy of Bavaria (from 788 part of the Frankish Empire) and the Avar Khaganate, the latter being situated in the Carpathian Basin (in today’s Hungary and the surrounding areas).^3^ The border between these two political units was, based on the study of written sources, the River Enns (situated today on the border of Upper and Lower Austria). The Avar Khaganate collapsed at the end of the 8th or in the early 9th century. In the 9th and 10th centuries, the area was divided among the following political entities: the Carolingian Empire and its successor states; the so-called ‘Great Moravian Empire’; the Přemyslid and the Slavnikid Chiefdoms; the Hungarian Chiefdom; and possibly the Bulgarian Empire as well as some other smaller polities. However, the extent of the territories belonging to these political units and their spatial changes through time cannot at present be reconstructed with great confidence. The principal spheres of influence are listed below in order to provide a basic framework of the political history of the area in the 9th and 10th centuries.

The Carolingian Empire and its successor states (first the Frankish Empire, then from 843 the East Frankish kingdom, ruled from 919 by the Ottonian dynasty) occupied today’s Austria south of the Danube, parts of W Hungary and possibly also certain regions of Austria north of the Danube. The occupation of the Frankish Empire and its successor states in regions east of the River Leitha (on the border of Lower Austria and Burgenland) lasted only until the Hungarian conquest in the late 9th century. For the region between the Rivers Leitha and Enns a Hungarian rule is postulated for the period 907–55, on the basis of written sources.

The so-called ‘Great Moravian Empire’ was situated from its formation in the 9th to its collapse in the early/mid-10th century in the SE part of today’s Czech Republic (Moravia), W Slovakia and NE Austria; in the late 9th century also in the W part of the Czech Republic (Bohemia) and possibly in parts of Hungary. The Přemyslid and the Slavnikid Chiefdoms were situated in the late 9th and in the 10th century in the W part of today’s Czech Republic (Bohemia). After the collapse of the ‘Great Moravian Empire’ the Přemyslid Chiefdom also occupied the E part of the Czech Republic (Moravia) and possibly parts of N and NE Austria.

The Hungarian Chiefdom was situated after the Hungarian conquest in the late 9th century and in the 10th century in today’s Hungary and in the surrounding areas. The Bulgarian Empire possibly occupied parts of E Hungary in the 9th century, before the Hungarian conquest.

The dynamic political history of the area from the 8th to the 10th century stands in surprising contrast to the characteristics of the fortified settlements and to elements of their material culture, which display strong similarities throughout the region. Some archaeological sites or groups of sites can be securely linked to one of the political units listed above, as for example the sites of Mikulčice, Břeclav-Pohansko and the settlement agglomeration in Uherské Hradiště and Staré Město can be connected to the so-called ‘Great Moravian Empire’, but this connection is based on their geographical location and not on the nature of the archaeological record.^4^

## THE POSITION OF FORTIFIED SETTLEMENTS IN THE LANDSCAPE

**Figure 2:**
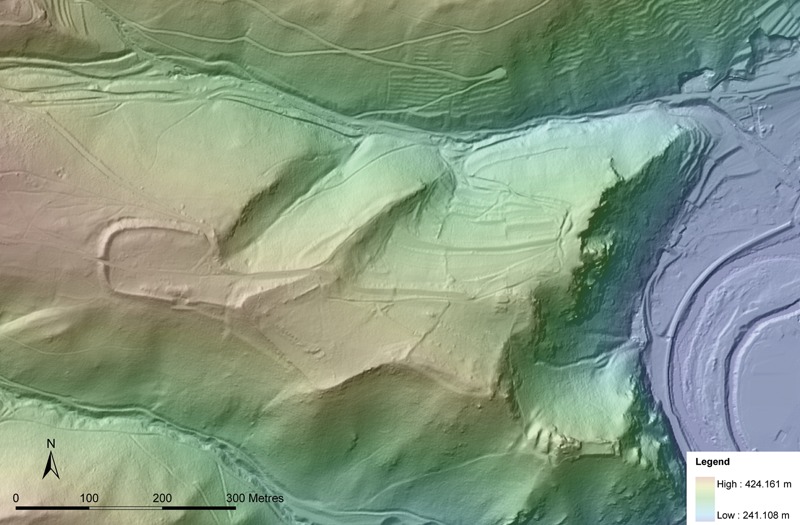
LiDAR scan of the fortified settlement of Gars-Thunau, Austria. On the left the fortification ramparts of the western part of the site (the entrance to the fortification in the west, visible on the LiDAR scan is of modern origin), in the centre the place of the manor farm, on the right the River Kamp and, in its valley, today’s village Thunau; the structures in the lower right corner, left of the legend, are remains of the 12th–13th-century castle ‘Schimmelsprung’. *LiDAR scan data from the Federal State of Lower Austria (Land Niederösterreich) 2009. Map by Christian Ansorge, Anja Masur and H Herold.*

**Figure 3:**
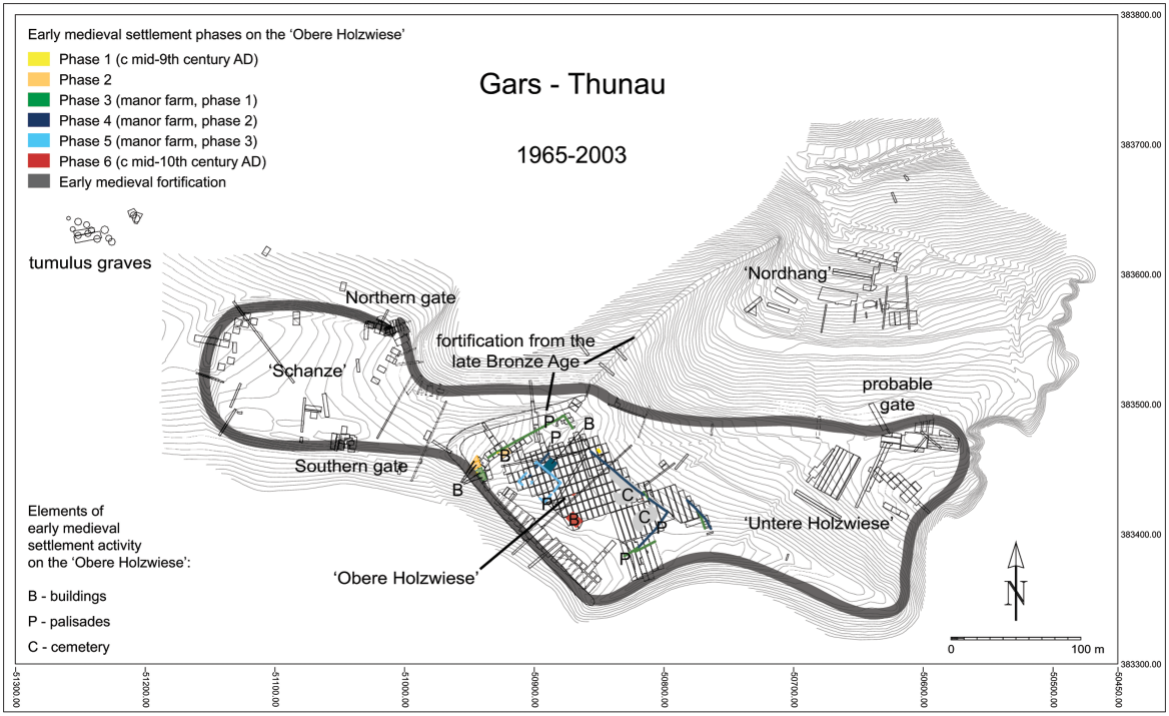
The fortified settlement of Gars-Thunau, Austria. Parts of the settlement, excavation trenches, gates, fortification ramparts and settlement phases. *Drawing by María Antonia Negrete Martínez (Department of Prehistoric and Medieval Archaeology, University of Vienna) and H Herold.*

There are two basic models for the position of fortified settlements in the landscape in 9th–10th-century central Europe: fortified hilltop settlements (eg Gars-Thunau [see Figs 2–3], Znojmo-Hradiště, Praha/Prague, Edelény-Borsodi földvár) and fortified ‘islands’, situated on sand dunes in (formerly) swampy areas in the floodplains of rivers (eg Mikulčice, Staré Město, Břeclav-Pohansko, Zalavár).^5^ The fortified hilltop settlements are usually smaller in size (c 0·5–12 ha) than the fortifications lying in floodplains (up to c 28 ha).

The location of the fortified settlements on hilltops or in floodplains surely depends on environmental parameters, but can also reflect cultural choices. Fortified settlements in floodplains are common in the 9th and 10th centuries, for example in SE Moravia and in the region of Zalavár (SW Hungary), while in Bohemia, in W Moravia and in NE Austria fortified hilltop settlements are typical.

From S Austria and from today’s Upper Austria hilltop settlements are known, but the available archaeological evidence for fortified settlements of the 9th and 10th centuries is limited in this region to surface finds and very small-scale excavations.^6^ In W Austria no fortified sites are currently known that can be securely dated to the 9th and/or 10th centuries. Fortified hilltop settlements were typical for Hungary, with a few exceptions.^7^ However, due to the lack of large-scale excavations, the dating of most of these sites to the 9th and/or the 10th century cannot be seen as reliable.^8^

Pobedim in W Slovakia illustrates how both types of fortifications can be simultane- ously present in one region.^9^ Pobedim is a fortified settlement lying in a floodplain; yet nearby are the fortified hilltop settlements of Ducové (c 10 km from Pobedim) and Bojná I, II and III (c 15–20 km from Pobedim).^10^ It is at present unclear why the founders of Pobedim and of the other sites chose different types of location for their fortifications.

In the case of the hilltop settlement of Gars-Thunau in NE Austria, the closest known fortifications from the 9th and 10th centuries (Sand near Oberpfaffendorf, c 30 km from Gars-Thunau; Znojmo-Hradiště, c 40 km from Gars-Thunau) are also hilltop settlements.^11^ From the 11th century onwards, however, fortifications in lower-lying areas are also known in the vicinity of Gars-Thunau (eg Sachsendorf; c 10 km from Gars-Thunau).^12^ This means that in principle both types of natural environments were available for building a fortification in this area, but the founders of Gars-Thunau decided to settle in a hilltop location. This seems to suggest that the geographical location of a fortified site is a result of a conscious cultural choice.

Other than the obvious differences in the location of fortified settlements on hilltops and in floodplains, no clear-cut differences are detectable in the structure of fortification ramparts, the type of buildings, site layout or elements of material culture between these two types of fortified settlements.

Remains of earlier occupation phases can frequently be found both at fortified hilltop settlements and at fortified settlements in floodplains (eg in Gars-Thunau and in Mikulčice). These settlement phases can originate from both prehistoric and historic periods. Usually multiple earlier settlement phases are present at the examined sites. There are, however, also fortified settlements which seem to have been built in previously uninhabited places (eg Edelény-Borsodi földvár). Many early medieval fortified hilltop settlements reuse late Bronze-Age (c 1000–800 bc) fortifications, in which case the fortification ramparts from the latter period were usually superimposed by an early medieval fortification rampart (eg in Gars-Thunau).

Some sites reveal early medieval occupations over late Bronze-Age fortifications, but without additional early medieval fortification ramparts. This is the case in Heidenstatt near Limberg and in Schiltern-Burgstall, both sites lying in the vicinity of Gars-Thunau in Lower Austria.^13^ Since in Gars-Thunau it seems that early medieval fortification ramparts were erected not at the onset, but rather in the course of the early medieval occupation, it is possible that a number of former late Bronze-Age fortifications had been reoccupied in the early Middle Ages, but only some of them were fortified again.

The question of how the 9th–10th-century population integrated remains of earlier occupation into the landscape of its own sites is, however, only one aspect of this phenomenon. Another, equally intriguing question regards their choice of previously occupied areas. Particularly in the case of former late Bronze-Age occupation, the earlier settlement phases must have been clearly visible for the early medieval population, since they yield very abundant surface material, especially ceramics. Since the number of favourable locations for settlement in the landscape was limited, it is logical that early medieval populations would have chosen to reoccupy strategically located late Bronze-Age settlements.

Another aspect is that (re)occupying the settlements of ‘ancestors’ can be used to legitimate one’s claims.^14^ Despite the vast chronological gap between these prehistoric settlement occupations and the early Middle Ages, elites could still have used such sites to legitimise their own political or territorial claims. The 9th and 10th centuries in central Europe were clearly a period of rapid social and political transition. Within this unstable socio-political climate, the need to legitimate claims of political authority and authenticity would have been real and powerful.

Thus, in addition to the representative character of a fortification itself as a sign of power and to the choice of the location of the site on a hilltop or in a floodplain, the existence of earlier occupation phases can be an important component of the symbolic meaning of the examined fortified settlements.

## SPATIAL ORGANISATION WITHIN THE FORTIFIED AREA

**Figure 4:**
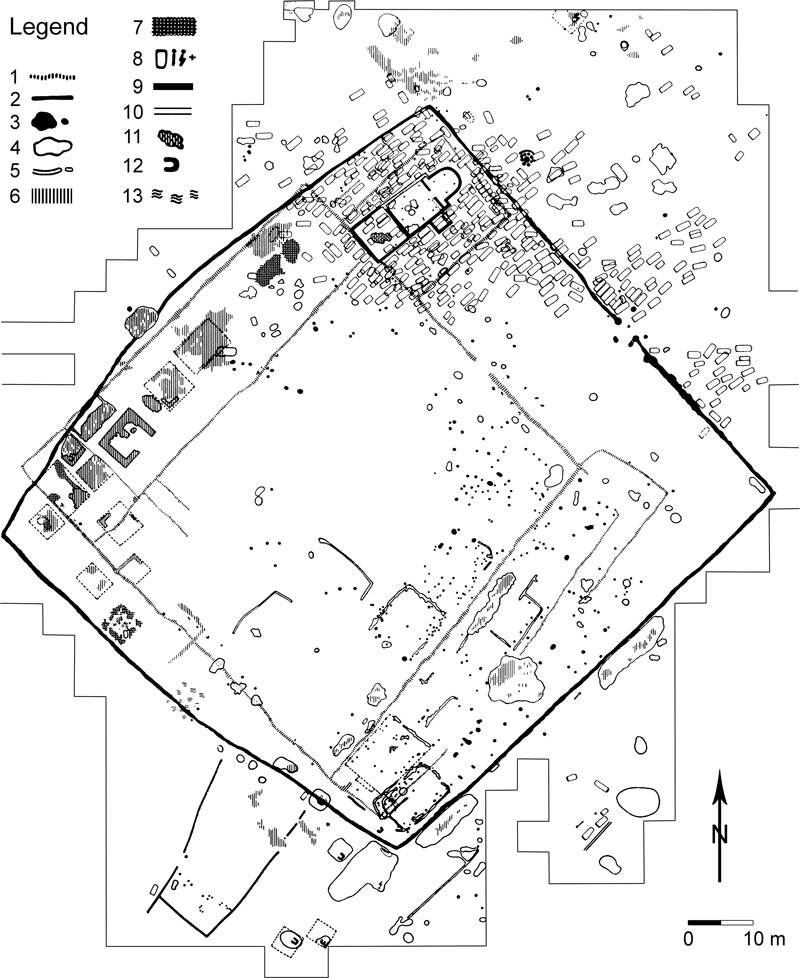
Břeclav-Pohansko, Czech Republic, ground plan of the manor farm. **1** Older phase of palisades. **2** Younger phase of palisades. **3** Postholes and small pits. **4** Outlines of larger buildings at ground level or with a sunken floor. **5** Foundation trenches of walls of buildings or of palisades. **6** Stone debris or stone platforms of buildings in log construction. **7** Fired marl plaster. **8** Inhumation graves with visible outlines of the grave pit (rectangles), inhumation graves without visible outline of the grave pit (schematic skeletons) and inhumation graves of which only a skull has been preserved (crosses). **9**, **10** Foundations of the church, foundation trenches with stones (9), foundation trenches with stones missing (10). **11** Collapsed wall of the church. **12** Ovens constructed of stones. **13** Concentration of finds at the place of settlement features that have no clear outlines. *After Dostál 1973, 300, Abb. 1, with modifications. Courtesy of 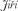 Macháček and Akademie Verlag.*

Some of the fortified sites — both fortifications in floodplains and on hilltops — include a ‘manor farm’ (*Herrenhof;* for the manor farm of Gars-Thunau see Fig 3). The manor farm was usually separated from the rest of the fortified area by a palisade. A church with a cemetery and one or more other buildings are often found within the palisade. Some of the buildings probably served as dwellings, others as ‘assembly’ or ‘reception’ halls (for more information on the buildings, see the sections below). The manor farms are usually interpreted as the separated living area of the ruling family of the site, which also had important symbolic functions in representing the high social status of these families. The manor farms vary in size between about 80×100 m (Gars-Thunau, Břeclav-Pohansko [Fig 4]) and 25×32 m (Zalaszabar-Borjúállás sziget) and can reflect the wealth and/or importance of the ruling family of that particular site.^15^ The size of the manor farm relative to the fortified area can, however, be quite varied: the manor farms of Gars-Thunau and Břeclav-Pohansko are similar in size, whereas the overall size of the two fortifications is markedly different (Gars-Thunau: 6 ha, Břeclav-Pohansko 28 ha).

The conceptual origin of ‘manor farms’ has not been fully explained yet. A connection to the Carolingian royal palaces (*Pfalz*) has been assumed, but similar structures have also been identified in Bulgaria.^16^ Moreover, the structure of (late) antique *villae* also displays some similar traits.^17^ In any case, the construction of structures similar to manor farms is not known from the regions under investigation here from the 7th to the mid-/late 8th centuries.

**Figure 5:**
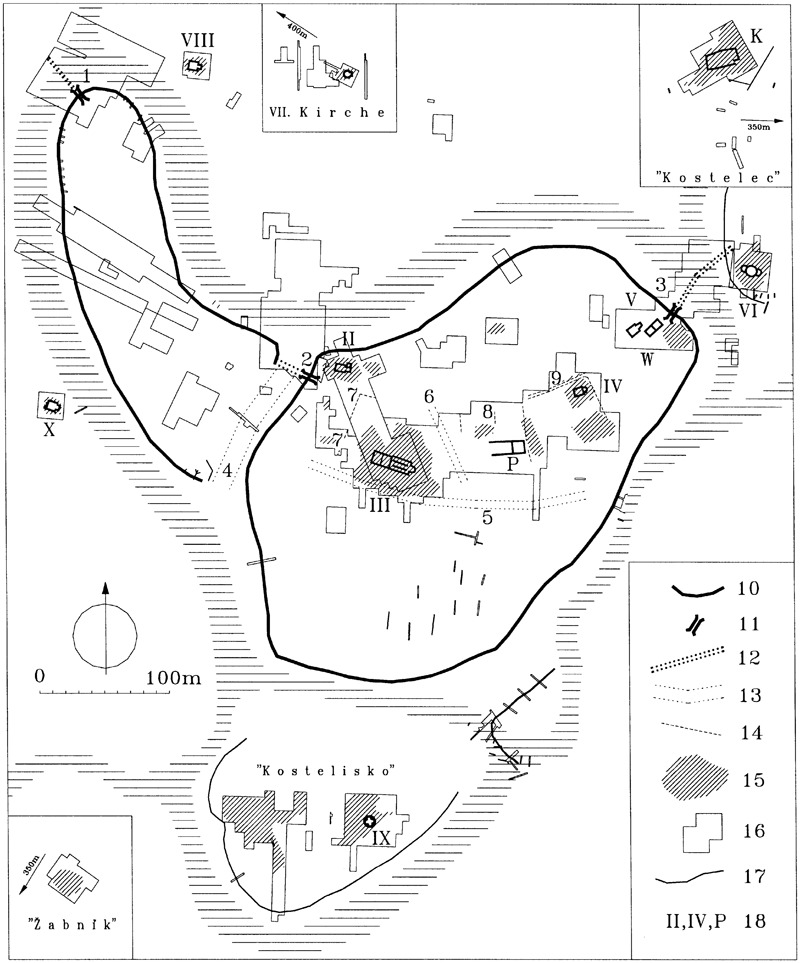
Mikulčice, Czech Republic, ground plan of the fortified settlement. **1** NW gate of the bailey. **2** Western gate of the acropolis. **3** NE gate of the acropolis. **4** Ditch between the acropolis and bailey. **5** Ditch south of church 3. **6** Ditch between the basilica and the ‘palace’. **7** Palisade wall of the area around the basilica. **8** Traces of palisade walls north of the ‘palace’. **9** Road and fence of the area around church 4. Legend: **10** Fortification ramparts. **11** Gates. **12** Bridges. **13** Ditches splitting the internal area of the fortified centre. **14** Fences and palisades inside the acropolis. **15** Burial places or significant groups of graves. **16** Investigated area. **17** Significant terrain edges. **18** Established numbering of churches (roman numerals), identification of the ‘palace’ on the acropolis (P), pagan temple in the place called ‘Klášteřisko’ (K) and jewellery workshop by church 5 (W); horizontal lines: arms of the River Morava. *Plan after Poláček and Marek 2005, 23, Abb. 7; figure caption after Poláček 2008c, 22, fig 9, with modifications. Copyright and courtesy of Lumír Poláček.*

**Figure 6:**
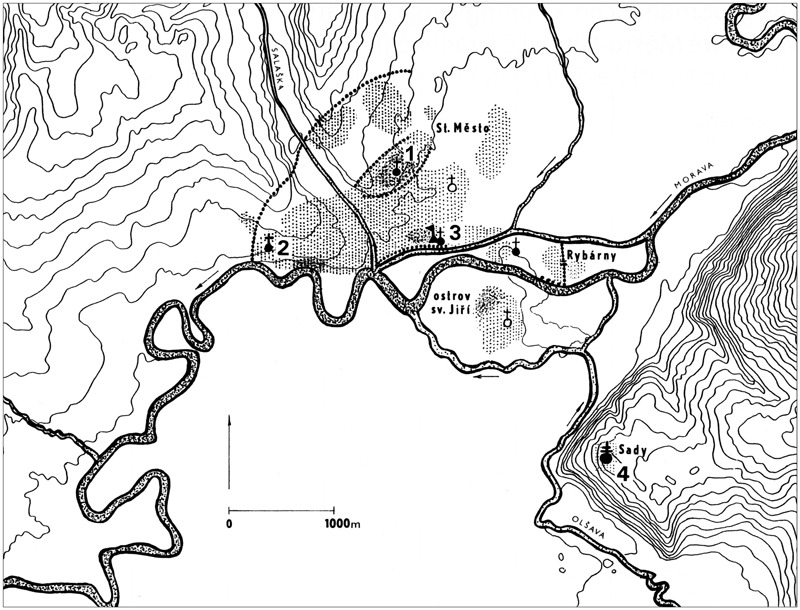
The settlement agglomeration in Staré Město and Uherské Hradiště, Czech Republic. **1** Area ‘Na Valách’. **2** Area ‘Na Špitálkách’. **3** Area ‘Na Dědině’. **4** Area ‘Sady’. Thick dotted areas: settlement areas before the 9th century; light dotted areas: settlement areas in the 9th and early 10th centuries; full circles with single crosses: excavated churches; empty circles with single crosses: assumed churches; large full circle with double cross: monastery; triangle with flag: ‘palace’ building; lines of large dots: fortification ramparts. *After Galuška 2008, 48, Abb. 23; figure caption complemented after Galuška 2006, 488, fig 2. Copyright and courtesy of Luděk Galuška and Universitätsverlag Wagner.*

**Figure 7:**
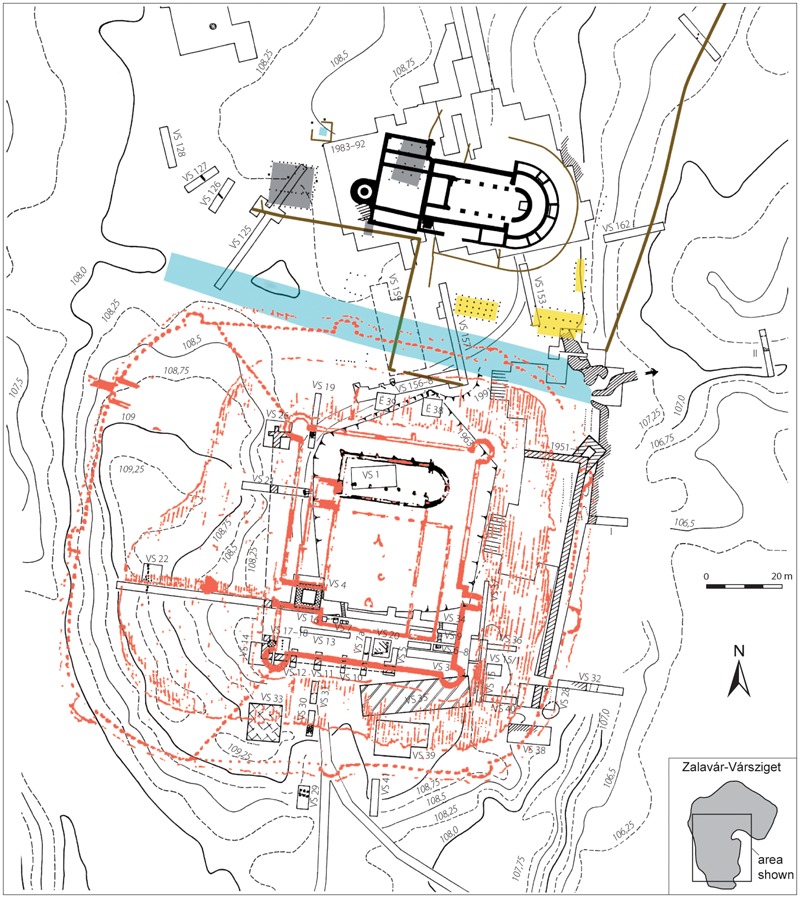
Zalavár-Vársziget, Hungary, southern part of the fortified settlement. Montage of excavation results until 1993 (excavators: Géza Fehér, Ágnes Cs. Sós) and since 1994 (excavator Béla Miklós Szőke) as well as of a drawing of Giulio Turco from 1569 (in red). Excavation trenches are indicated until 1993. Black: remains of churches built in stone (the northern church has been excavated, the southern church is only known from the drawing of Giulio Turco); grey: excavated remains of a church and a possible monastery building built in wood; yellow: excavated remains of other buildings built in wood; brown: excavated and reconstructed course of palisades; blue: trench separating the southernmost part from the rest of the ‘island’ and a well (baptistery?); area filled with cross grid: excavated remains of a tower built in stone; area filled with black diagonal and horizontal lines: excavated remains of fortification (11th century?); area filled with dense black diagonal lines: excavated remains of fortification (9th century?). *After Szőke 2009, 404, Abb. 5, with modifications. Copyright and courtesy of Béla Miklós Szőke and Habelt Verlag. Figure caption by H Herold.*

**Figure 8:**
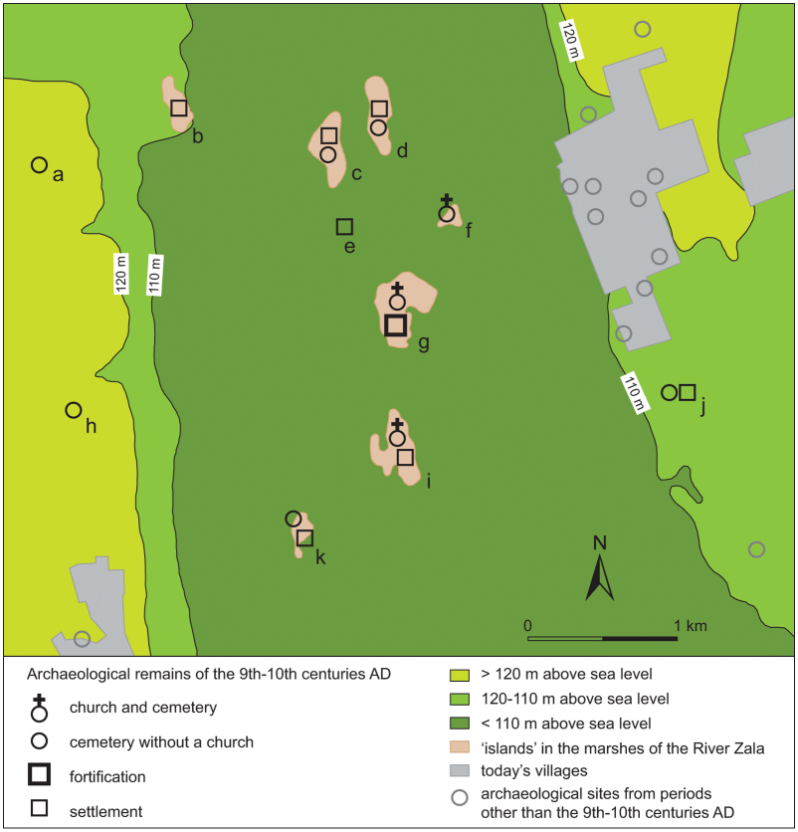
Settlement agglomeration near Zalavár, Hungary. **a** Esztergályhorváti-Alsóbárándpuszta. **b** Esztergályhorváti-Huszárvár. **c** Zalavár-Rezes sziget. **d** Zalavár-Kövecses sziget. **e** Zalavár-Zalapart. **f** Zalavár-Récéskút. **g** Zalavár-Vársziget. **h** Zalaszabar-Kisesztergály. **i** Zalaszabar-Borjúállás sziget. **j** Zalavár-Mekenye. **k** Zalaszabar-Dezső sziget. *Map by H Herold, redrawn and adapted after Szőke 2007b, 835–6, Abb. 107–8; Szőke 1992a; Költ*ő *and Vándor 1996, map in appendix; Cs. Sós 1984, 157, Abb. 1 and Müller 1992, 327, Abb. 26.*

Some sites, such as at Gars-Thunau and Břeclav-Pohansko, include only one manor farm, which appears to be the centre of these sites. More complex sites, such as Mikulčice (Fig 5), the settlement agglomeration in Uherské Hradiště and Staré Město (Fig 6) or Zalavár (Figs 7–8), have multiple parts, which include more churches and possibly also more ‘manor farm’ units. These can be different in size, which probably indicates differences in their importance. The varied complexity of the internal structure of the investigated fortified settlements can be indicative of the functions and symbolic meaning that these sites possessed.

The areas of the fortifications lying outside of the manor farm(s) but within the fortification are usually connected to craft production. A large number of craft activities are present in these areas; among them ironworking seems to play a very prominent role. Using geomagnetic prospection in Břeclav-Pohansko, a system of rectangular structures was detected within the fortified area but outside of the manor farm. These were interpreted as individual plots of land. In Zalaszabar-Borjúállás sziget a row of plots seems to have existed before the small manor farm was built at the site. However, these cases are so far unique in the region.

There are also sites that do not include a ‘manor farm’. An example of such sites is Mužla-Čenkov in S Slovakia, on the N bank of the River Danube.^18^ At this site mainly sunken-featured buildings were excavated within the fortified area and the fortification did not comprise a church.

Some of the discussed fortified sites display a complex system of ‘suburbia’ or baileys, areas adjacent to the main fortification (eg Břeclav-Pohansko and Mikulčice). These can be fortified, but their fortification ramparts are usually on a smaller scale than those of the main fortification. A possible function of these areas, on the basis of the current available archaeological evidence, might have been to house the military entourage of the ruling family of the fortification.

## CHURCHES IN THE FORTIFIED AREA

The best-preserved building remains in the early medieval fortified settlements of central Europe are the remains of churches. These buildings were the first ones to be built in stone, although remains of wooden churches have also been excavated. The site of Mikulčice had an unusually high number of early medieval churches: 12 buildings were identified as churches during excavation, although now only nine are thought to have actually served such a purpose. In most cases only the foundations of the churches have been recovered through excavation. However, the small and still-standing stone church at Kopčany (Slovakia) near Mikulčice is a notable exception, which has been dated to the 9th century based on its wall structure and the accompanying graves.^19^ In the settlement agglomeration of Staré Město and Uherské Hradiště, four stone churches have been excavated (Staré Město in the areas ‘Na Dědině’, ‘Na Valách’, ‘Na Špitálkách’ and in Uherské Hradiště-Sady).

A group of 9th–10th-century churches came to light in the surroundings of Zalavár. The main site of this settlement agglomeration is Zalavár-Vársziget. Here excavators found a church of 30 m length, built in stone and having an ambulatory crypt. Within the same settlement a rectangular wooden building (c 12×12 m) has also been identified as a church.^20^ Some 600 m north-east of Zalavár-Vársziget, another stone church (c 20×12 m) has been recovered on a nearby sand dune, in Zalavár-Récéskút. On another neighbouring sand dune, on Zalaszabar-Borjúállás sziget, a wooden church (c 17×7 m) has been found. The latter church probably had a sill-beam construction.

In Gars-Thunau the foundations of a small stone church have been found, but because of the absence of early medieval graves it cannot be securely dated to the 9th–10th centuries. It is also probable that at the same site a small, wooden church was situated within the 9th and 10th-century cemetery. In Wieselburg a still-standing octagonal church of the 10th century has been integrated into the modern church building. This site is associated with the place *‘Zuisila’* mentioned in the written sources and was most probably fortified in the later phase of the early Middle Ages, but no modern excavations have been carried out to determine the structure and the age of the fortification.^21^ Although a number of other still-standing churches with possible 9th–10th-century predecessors can be found in Austria, there is no archaeological evidence to suggest the presence of associated contemporary fortified settlements.^22^

Concerning the layout of the stone churches in the examined fortified settlements, the smaller churches (up to about 15–17 m in length) belong to the following basic types:

- round churches with one or more apse(s) (eg Ducové; Mikulčice, church 6; Prague, church of St Vitus);
- rectangular churches with a rectangular/trapezoidal choir (eg Mikulčice, churches 5, 8, 10; Kopčany; Staré Město, area ‘Na Valách’);
- rectangular churches with an apse (eg Mikulčice, church 4; Staré Město, area ‘Na Špitálkách’; Edelény-Borsodi földvár).

There are a large number of variations in the floor plans of these small churches. Larger churches are relatively rare (eg Mikulčice, church 3; Zalavár-Vársziget, church with ambulatory crypt; Zalavár-Récéskút); they all have an individual ground plan.

Historical sources indicate that missionaries from both the Byzantine and the Frankish Empire converted the area of central Europe under consideration here in the 9th and 10th centuries ad. The Byzantine mission included most importantly the activities of Methodius, his brother Cyril and their disciples. From the Frankish Empire, the Archbishopric of Salzburg, the Bishopric of Passau, and possibly also the Patriarchate of Aquileia were active in converting the population.

The different ground plans of churches from the fortified sites reflect this overlapping missionary activity; they show similarities to churches in the Frankish Empire (including both parts of today’s Germany and Italy) as well as in the Byzantine Empire.^23^ It is, however, difficult to establish clear connections between any one of these buildings and one certain missionary centre from the Carolingian/Ottonian or Byzantine areas. It is thus not realistic to reconstruct activity areas of the different missionary centres on the basis of the archaeological record. In addition, it is probable that the dimension and location of missionary activities from the different Christian centres shifted in the course of the 9th and 10th centuries.

A few structures at some of the fortified sites may have been connected to ‘pagan’ ritual activity. Two structures in Břeclav-Pohansko, each consisting of nine large posts situated very close to each other, were excavated and are interpreted as ‘pagan sanctuaries’. They are supposed to postdate the flourishing period of the fortified settlement and are dated to the 10th century. One structure at Mikulčice, possibly representing the remains of a building, has been interpreted as related to ‘pagan’ cult activities. Its relative date compared to the other excavated features at the site has not yet been clarified. If the interpretation of these remains as being connected to ‘pagan’ ritual activities is correct, it indicates that, in addition to the overlapping missionary activities of the Christian Church mentioned above, other ritual backgrounds (and connected political forces?) were present in 9th–10th-century central Europe.

## OTHER BUILDINGS IN THE FORTIFIED AREA

Only in a very few cases can we assume the existence of stone buildings other than churches at the fortified sites. These include the ‘palaces’ of Mikulčice (c 11× at least 26 m; the position of the W end of the building is not exactly known) and Staré Město (in the area ‘Na Dědině’; c 10× at least 19 m; the position of the E end of the building is not exactly known), which are usually reconstructed with stone walls.^24^ These buildings are likely to have been large ‘assembly’ or ‘reception’ halls and to have had important symbolic functions in representing the high social status of the leading families of the sites. A building (c 7·5×6 m) with a stone foundation has been excavated at Břeclav-Pohansko. It is, however, usually reconstructed with wooden walls. Stone foundations of a building of c 5×5 m have also been uncovered at the site of Edelény-Borsodi földvár. It is not completely clear if this building was made entirely of stone or if it possessed a stone foundation with wooden or wattle-and-daub walls.

Most other known buildings at these sites seem to have been constructed in wood, or with wattle-and-daub walls. Some of them, especially larger constructions, like the buildings in Stará Kouřim (89×10 m), Levý Hradec (approximately L-shaped, 15×5 and 10×5 m) and Uherské Hradiště-Sady (L-shaped, 40×10 m and 10×10 m), might have served as ‘assembly’ or ‘reception’ halls, similar to the large stone buildings mentioned above.^25^

Smaller buildings in post-construction have been excavated at some sites (eg Břeclav-Pohansko, Gars-Thunau). In Gars-Thunau buildings of a similar dimension in log construction seem to have existed as well. The buildings of both construction types are likely to have been placed at ground level. However, as the original floor layers contemporary with the buildings have been destroyed, the possibility of a slightly sunken floor cannot be excluded. The size of these houses is variable, but a common mean size is about 8–9 m×4–5 m. At present no internal division of these buildings can be shown. Similar buildings in log construction placed at ground level, or possibly having a slightly sunken floor, were excavated at the site of Edelény-Borsodi földvár, but their size is considerably smaller (c 4×4 m). It is difficult to reconstruct the function of these buildings of various sizes with wooden and/or wattle-and-daub walls, but most of them included hearths/ovens and it is probable that some of them served as dwellings.

Barns or other storage buildings as well as byres or stables cannot at present be securely identified in the archaeological record of the discussed fortified settlements.^26^ The remains of the buildings in Edelény-Borsodi földvár mentioned above suggest that the houses at this site included a separated roof space, apparently used for storing grain. From other settlements the only securely identifiable storage facilities are the (mostly cylindrical) pits for storing grain.

The most common type of building in the contemporary unfortified, open settlements of the region is the so-called sunken-featured building (*Grubenhaus*). These seem to have been used mainly as dwellings, in contrast to W Europe during the same period. Sunken-featured buildings are relatively rare in the central areas of most fortifications in central Europe.^27^ In the ‘suburbia’ of some fortified sites (eg Břeclav-Pohansko) sunken-featured buildings are present in large numbers. It is currently unclear whether this difference simply indicates a difference in social rank or if it is connected to other factors.

Pits of different shapes, and at some sites also wells, complement the types of settlement features excavated in fortifications of the 9th and 10th centuries in the study area. While cylindrical pits for storing grain are present at most sites, other types of pits exhibit a wide range of variability among fortified sites; such differentiation probably largely depended on the geographical position of the sites (sites on sand dunes in swampy areas vs hilltop settlements with rocks not far under the surface). Wells in some cases contain a timber construction, which is important for dendrochronological dating, such as at Břeclav-Pohansko.^28^

## FORTIFICATION RAMPARTS

The fortifications examined in the present article were typically fortified by ramparts made of a combination of wood, stones and earth. The basic element of the fortification ramparts is a timbered caisson (box) construction made of wood and filled with earth or with a mixture of earth and stones. Sometimes a layer of timbers (lying perpendicular to the line of the wall or crosswise) beneath the caisson construction serves as a foundation. This construction can have a facing wall on the exterior or on each side, but there are also fortification ramparts without a facing wall. The facing wall can be made of quarry stones of different types; at many sites stones naturally breaking into flat pieces were preferred (eg granulite in Gars-Thunau).

The construction of the ramparts (size, shape and number of the timbered caissons, existence and types of the facing wall(s) etc) was accomplished in a number of different ways. Rudolf Procházka collected 13 basic variants of the caisson construction from the E part of the Czech Republic.^29^ In some cases, a single fortification employed more than one of these variants. At some sites ramparts made only of earth were excavated (Procházka Type 3); it is not clear whether these represent a separate construction-type, or if the preservation of wood was so poor at these sites that the remains of the caisson construction were not recognised.

Currently no secure chronological or geographical distribution patterns have been established for the various types of fortification ramparts with caisson construction. This is partly because many large-scale excavations are still unpublished and are thus difficult to date. Another reason is that in a number of fortifications only the ramparts were excavated and they are not typically datable on their own.

The dendrochronological dating of 9th–10th-century wood samples became possible in the examined region by the end of the 1990s, largely based on the work of the late Jitka Vrbová-Dvorská. Dating the wooden caisson construction of fortification ramparts holds much promise for precisely dating the discussed fortified sites. However, so far few dendrochronological dates have been published. Furthermore the waney edge (the last growth ring of a tree, *Waldkante*) has usually not been preserved, so even if there are dendrochronological dates, they provide only a *terminus post quem* in most cases, not the exact year when the tree was cut. Another problem is establishing the chronological sequence of the fortification rampart and of other excavated features within the fortifications, as in most cases no stratigraphic sequence of these exists.

Dendrochronological dates from fortification ramparts are available from Gars-Thunau (ad 834–94, 48 samples, all samples without a waney edge).^30^ This data shows that the fortification was probably built after ad 830 and that building activity was ongoing at the end of the 9th century. The nature of the available data and its spatial distribution within the site does not indicate whether its ramparts were built in a single or multiple building phases.

Dendrochronological dates exist for Mikulčice, Břeclav-Pohansko and Znojmo-Hradiště, in Mikulčice primarily from parts of bridges and from wooden constructions against erosion.^31^ The dates from Mikulčice cover the period ad 737–872 (in most cases without a waney edge).^32^ As mentioned above, the timber structure of a well within the fortified area of Břeclav-Pohansko was dendro-dated to ad 882. One sample from the fortification of Břeclav-Pohansko yielded a date of ad 875 and one sample from the fortification of Znojmo-Hradiště dates to ad 888 (both samples without a waney edge).^33^ We await the publication of dendro-dates from different fortified sites of the 9th and 10th centuries in Slovakia.^34^

Dendrochronological dates from Prague span the period from ad 845 to 958 (in most cases without a waney edge). The youngest dates from the oldest fortification ramparts of the Prague castle are ad 906–17 (without a waney edge).^35^

Only in very few cases were freestanding, dry masonry walls observed in the fortifications of the examined region.^36^ At most of these sites both freestanding dry masonry walls and ramparts made of wood, stones and earth, with facing walls have been excavated. This was the case in Sand near Oberpfaffendorf (Lower Austria), where timbers were used as the foundation of the facing walls of the ramparts. Dendrochronological dating of these timbers yielded a date of ad 926–30 (samples with a waney edge).^37^ This date possibly means that in this part of the examined region freestanding, dry masonry walls appear at a somewhat later chronological stage than the fortification ramparts with caisson construction. However, this hypothesis has to be verified by a larger number of dendrochronological dates from more sites.

Scientific dating, including radiocarbon dating of animal and human bones, is sure to play an increasingly important role in dating the discussed sites in the upcoming decades, concerning both the fortification ramparts and their chronological relation to the settlement features and graves in the fortified area.

## GRAVES WITHIN THE FORTIFICATIONS

Single graves, groups of graves, or larger cemeteries have all been identified alongside the remains of settlement activity at most of the fortified settlements examined here. They were in most cases not fully separated from the habitation area by archaeologically visible boundaries. Palisades often surrounded one or more side(s) of cemeteries, particularly in the manor farms, but graves are also found outside of the palisades at virtually every site. In some cases, the location of graves above the foundation trench of the palisade indicates that the cemetery continued to be used after the palisade was removed (eg Zalaszabar-Borjúállás sziget). At other sites graves are found both inside and outside of the palisade, but do not clearly superimpose the foundation trench of the palisade (eg Gars-Thunau).

Single graves or small groups of graves, comprising two to three individuals, were placed throughout the fortified area; they are frequently found in the vicinity of fortification ramparts, but can also occur without a clear connection to other excavated features. The graves can predate, postdate or be contemporary with the fortification ramparts. Single graves in the areas under discussion here usually do not contain any gravegoods, although simple objects, like iron knifes, are occasionally present. It is probable that at least a part of these single graves or small grave groups represent ‘special deposits’.^38^ Their deposition can be connected to the beginning or ending of different phases of building activity, or can indicate a special function of a part of the fortification (eg fortification ramparts signifying a border).

Larger grave groups of c 7–15 graves can be found at different points within the fortified settlements (eg in Břeclav-Pohansko). These grave groups are interpreted as belonging to smaller habitation units of the site, perhaps correlated with the ‘plots’ of land detected by prospection methods. These graves seldom contain gravegoods; if they do, they comprise mostly simple items.

Most of the discussed sites include one or more larger cemeteries (>50–100 graves) situated around a church. Some of these cemeteries and churches are parts of a manor farm (eg Břeclav-Pohansko, Gars-Thunau, Zalaszabar-Borjúállás sziget). In other cases only the church and the cemetery have been excavated and information on the further surroundings is not available (eg Staré Město, area ‘Na Valách’; Mikulčice church 6). Several graves within the cemeteries around churches contain very rich gravegoods, most of which can be considered as part of the ‘dress’ and ‘equipment’ of the deceased. These gravegoods can include jewellery (eg earrings, necklaces of beads, brooches, dress needles), parts of belts (eg buckles, belt fittings, strap ends, parts of garter belts), weapons (eg swords, axes, lances, arrowheads, parts of mail shirts) and tools for craft production (eg sewing needles, awls). Similar objects occur among the finds of the settlements as well, but in much smaller numbers.

In exceptional cases cemeteries connected with churches also included graves within the church building.^39^ The very small number of these graves and the graves of identified members of the ruling family in Prague make it probable that this form of burial was restricted to the highest elites. The known graves from churches were, however, not as rich in gravegoods as some graves known from the vicinity of churches. This can be a result of a different way of expressing wealth in the leading families, either through archaeologically hardly visible objects in the graves, like garments made of expensive textiles (as shown eg for the grave of Boleslav II in Prague) or through donations to the Church (by building a church or in other ways).

The graves in the examined fortified settlements, especially those in cemeteries around churches, very rarely contain ceramic vessels. The occurrence of ceramic vessels in many cemeteries is restricted to graves of children. Animal bones are found very seldom in graves within fortified settlements; they are, however, similarly to ceramic vessels, present in cemeteries of open settlements in the same region.^40^ These differences indicate that the self-presentation of most inhabitants of the fortified sites included other elements than that of the population living in open (mainly agrarian) settlements. However, the symbolic meaning of these elements cannot be exactly reconstructed.

Graves of animals are very rare at the discussed fortified sites. Graves of horses were found in Břeclav-Pohansko and in Mikulčice, and are believed to be connected to the ‘pagan sanctuaries’ excavated at these sites.

## CONNECTIONS TO THE SURROUNDING LANDSCAPE

A network of agrarian settlements must have surrounded the examined fortified settlements. However, archaeological work has largely concentrated on the (very costintensive) research of the fortified sites themselves and less effort has been invested into the research of the surrounding area.^41^ Most agrarian settlements near fortified sites are only known because of surface finds from archaeological surveys or from small-scale excavations. Some cemeteries have also been excavated in the surroundings of the fortified sites, a part of which must have belonged to agrarian settlements. However, it is rather difficult to establish a chronological connection between the agrarian and the fortified sites; ie to determine which agrarian sites are actually contemporary with each other and with the fortification.

Larger fortified settlements in particular are surrounded by a network of sites that go beyond the level of agrarian settlements, and include different branches of craft production and can comprise a manor farm and/or a church. These surrounding sites are in many aspects similar to the main fortification, only smaller in size. Such networks existed both for fortified settlements on hilltops (eg in Nitra) and in floodplains (eg in the surroundings of Zalavár, Fig 8).^42^

## CONCLUSION

The sites discussed in the present article have different origins, but they nevertheless developed into very similar fortified sites in the course of the 9th and 10th centuries ad. These were the first early medieval settlements in the examined region, excluding former Roman fortresses with early medieval occupation, which remained in a fixed location during their entire existence and were not regularly shifted in the landscape. Many of the discussed sites have also been rebuilt a number of times on the same spot, a phenomenon that underlines the importance of the sites’ location to their inhabitants.

The density and size of fortified settlements are highly varied throughout different parts of the investigated region.^43^ This uneven intensity of settlement activity in different areas can reflect (certain aspects of) the distribution of political power. The estimated number of inhabitants at the investigated sites ranges from 50–100 at the smaller sites, like Gars-Thunau, ‘at least 700’ for Břeclav-Pohansko, to c 3000–4000 at larger sites, like the settlement agglomeration at Staré Město and Uherské Hradiště.^44^ These figures and the uneven geographical distribution of larger and smaller fortified sites in 9th–10th-century central Europe indicate, even if one takes into account their inherent uncertainties, that in the various sub-regions of the investigated area the density of population is likely to have been very different.

It is a matter of debate if the inhabitants of the discussed fortified settlements took part in agrarian production themselves or if they (fully or partly) relied on agrarian products from surrounding smaller unfortified settlements. Evidence from Gars-Thunau suggests that animals were kept in or around the fortified settlement and that no large-scale import or export of animals or parts of animals can be assumed.^45^ It has been supposed that the population of Břeclav-Pohansko relied to a great extent on external sources for agrarian products.^46^ Comparative studies including additional sites could potentially shed more light on this issue. A large number of craft activities has been detected at most of the investigated fortified sites, but evidence for large-scale ‘industrial’ production, similar for example to that of the *emporia* in the North Sea zone and around the Baltic Sea, has to date been missing.^47^

Indeed, in the investigated region no settlements have been excavated that are similar to *emporia* or ‘ports of trade’.^48^ Certain aspects of *emporia*, like (apparently) planned streets have been excavated at some sites, but the overall structure of the sites is markedly different. The written sources mention a ‘market of the Moravians’ and describe trade with different items (wax, salt, animals, slaves, etc).^49^ However, the marketplaces and the traded goods have not been yet identified in the archaeological record of the investigated region. Coin finds in the examined area include Byzantine, Arabic and Frankish coins, but they are exceptionally rare and can probably not be directly connected to trade or to a monetary economy.^50^

The presence of churches at practically all of the examined fortifications emphasises the importance of these sites in a period and region where churches are exceptionally seldom known from other contexts. The fortifications are likely to have served as ‘gateways’ for the Christian conversion of the population, probably starting with the leading families of these sites and followed by the groups of people dependent on them. For Uherské Hradiště-Sady and Bojná it has been considered that these sites entirely belonged to the church and served as ‘bases’ for the conversion activities. In addition to the competition of different Christian centres in the conversion of the area, possible ‘pagan sanctuaries’ at Břeclav-Pohansko and Mikulčice indicate that this process was carried and at times probably also obstructed by a number of different forces.

Fortifications similar to the ones examined in this article are also known from different parts of Germany and Poland.^51^ It seems likely that fortifications with a rather simple layout and usually of smaller size, like at Gars-Thunau, were the seats (or one of the seats) of distinguished families. Thus they are likely to have served as centres for their surroundings concerning economic, administrative, defensive and ritual/religious matters. It is indeed very probable that the emergence of the discussed sites in the 9th and 10th centuries reflects the reorganisation of elites in the regions concerned. It is interesting to note, however, that the representation of these reorganised elites follows similar principles in the investigated border regions of the Frankish Empire and its successor states both on the interior and on the exterior of the Empire’s political border.

The more complex sites, such as Mikulčice and possibly also Staré Město, appear to represent a different type of settlement concept, which has to date not been identified in the Frankish Empire or its successor states. The central unit of a ‘palace’ and of a large church (church no 3) at Mikulčice are surrounded by units made up of smaller churches and possibly of other buildings. This spatial arrangement can perhaps be interpreted as the central representative unit of a ruler and surrounding smaller units, belonging to other ‘noble families’ dependent on the ruler. These ‘noble families’ might have had their main seat(s) elsewhere. Certainly, this interpretation has to be verified by further research; the described spatial structure nevertheless exists and is different from settlement concepts known from the Frankish Empire and its successor states.

The sites discussed in the present article were contemporaries of Hedeby/Haithabu, York (Eoforwic/Jórvík) or San Vincenzo al Volturno and cover the dynamic period when many *emporia* in W Europe, like Hamwic or Dorestad, were abandoned and early towns came into existence in their vicinity.^52^ This paper has aimed at giving a short overview of 9th–10th-century fortified sites in today’s Austria, Hungary, Czech Republic and Slovakia. An increasing interest in this area and the integration of research results on the discussed sites into a wider geographical context can aid a more complete understanding of the early Middle Ages in Europe.

## ABBREVIATIONS IN BIBLIOGRAPHY

AV ČR: Akademie Věd České Republiky [Academy of Sciences, Czech Republic]
Antaeus: Communicationes ex Instituto Archae- ologico Academiae Scientiarum Hun- gariae, Budapest

## Footnotes

2 Such an overview concentrates on main traits and the most important characteristics of these sites, based primarily on sites where a substantial amount of archaeological evidence is available. It is not intended and also not possible here to mention every site that was, or might have been, a fortified settlement in this period; a number of sites, especially those with only surface finds or with small-scale excavations, must remain unmentioned.

3 For the political history of the region from the 8th to the 10th century, see also Pohl 1988, Wolfram 1995 and Brunner 1994.

4 Mikulčice: Poláček 2002; 2007. Břeclav-Pohansko: Macháček 2007 (published also in English as Macháček 2010). Uherské Hradiště and Staré Město: Galuška 2005; 2008. In order to maintain clarity in the present article general references to the examined sites are provided only at first mention. As general references the most recent publications are named; these usually also include references to earlier works. If referring to a special aspect of a site, especially if separate publications are available on that particular topic, further references are given.

5 Gars-Thunau: Herold 2008. Znojmo-Hradiště: Klíma 2001. Praha/Prague: Frolík 2002. Edelény-Borsodi földvár: Wolf 1996; 2001. Zalavár: Szőke 2007a; 2007b.

6 Felgenhauer-Schmiedt 1993; Gutjahr 2006; Trebsche 2008.

7 Such exceptions are eg Zalavár, mentioned above, and the fortification of Szabolcs in NE Hungary (Fodor 1996, 433–4).

8 Thus in the present article from the area of today’s Hungary only the extensively excavated fortified settlements in the region of Zalavár and at the site of Edelény-Borsodi földvár are going to be discussed.

9 Bialeková 1998.

10 Ducové: Ruttkay 2005. Bojná I, II and III: Ruttkay 2009. All distances between sites are given as the crow flies.

11 Sand near Oberpfaffendorf: Felgenhauer-Schmiedt 2008.

12 Krenn 1991.

13 Heidenstatt near Limberg: Tuzar 1998. Schiltern-Burgstall: Trnka 1981.

14 Bradley 2002; I am grateful to Helena Hamerow for drawing this possible interpretation to my attention.

15 The term ‘manor farm’ is used in this article as a technical term in the place of the German word *Herrenhof*, as there is no established expression for these central European structures in English. A direct translation of *Herrenhof* would be ‘lord’s farm’. In Western Europe the term ‘manor farm’ is used to mean a bipartite estate. At the current state of research it is not clear if the *Herrenhof* structures in central Europe were bipartite estates. Zalaszabar-Borjúállás sziget: Müller 1995.

16 Carolingian royal palaces (*Pfalz*): eg Dostál 1975; Ruttkay 1998; Macháček 2001. Bulgaria: Henning and Milo 2007.

17 Brather 2008, 110.

18 Hanuliak et al 1993.

19 Poláček 2009.

20 Szőke 2009.

21 Only preliminary reports of the small-scale excavations of the 1950s are available from the site (Ladenbauer-Orel 1996). On the written sources see Brunner 1994, 96.

22 For information on these churches see different contributions in Sennhauser 2003.

23 Poláček 2008a; 2009; Galuška 2005.

24 Only the stone foundations of these buildings were archaeologically documented. The possibility that their walls were made of wood cannot be completely ruled out.

25 Stará Kouřim: Šolle 1966. Levý Hradec: Borkovský 1953. Uherské Hradiště-Sady: Galuška 2005, this latter building is usually interpreted as the main building of a monastery.

26 The analysis of animal bones from Gars-Thunau indicated that livestock were kept in or in the vicinity of the fortified settlement (Kanelutti 1990), thus it is likely that byres and/or stables existed in this area. However, these buildings/structures cannot at present be identified in the archaeological record.

27 With the notable exception of the site of Mužla-Čenkov (Slovakia) that comprised 66 sunken-featured buildings on the excavated area of 4600 sq m.

28 The timber construction of a well within the fortification of Břeclav-Pohansko was dated to ad 882 (Macháček 2007, 48).

29 Procházka 2009, 11, Abb. 1:4–16.

30 Cichocki 1999.

31 Dvorská et al 1999, 72, Abb. 3.

32 Poláček 2005, 14.

33 Břeclav-Pohansko: Dresler 2008, cited in Grabolle 2008, 39. Znojmo-Hradiště: Dresler 2004, cited in Grabolle 2008, 40.

34 Pers comm Joachim Henning, February 2011.

35 Dvorská and Boháčová 1999, 58–9, Abb. 3.

36 The screen walls of the ramparts in caisson construction are also dry masonry walls, but they constitute a part of the rampart and do not stand on their own.

37 Grabner 2002, cited in Felgenhauer-Schmiedt 2008, 308.

38 The term ‘special deposits’ is used here according to Hamerow 2006.

39 Schulze-Dörrlamm 1993.

40 Eg in the vicinity of Zalavár in the cemeteries of Garabonc-Ófalu 1 and 2 (Szőke 1992b).

41 Available results on this topic are discussed in Poláček 2008b.

42 Nitra: Fusek and Bednár 2008.

43 See eg Kouřil 2009, 360, Abb. 1.

44 Břeclav-Pohansko: Macháček 2007, 426. Staré Město and Uherské Hradiště: Galuška 2005, 527.

45 Kanelutti 1990.

46 Macháček 2007, 48.

47 For the term ‘*emporia*’, see Hodges 1982. For large-scale craft production in *emporia* see eg Westphalen 1989; 2002; Ulbricht 1978; all three works on Hedeby/Haithabu.

48 For the term ‘ports of trade’, see Polanyi 1957.

49 The data originates from the ‘Raffelstetten inquest’; Mitterauer 1980, 235–77.

50 McCormick 2001, 348, map 12·1 and 371, map 12·6; Kovács 1989; Hahn 1990; Kučerovská 1998.

51 Germany: eg Ettel 2001. Poland: eg Poleski 2004.

52 Hedeby/Haithabu: eg Jankuhn 1986, von Carnap-Bornheim and Hilberg 2007. York (Eoforwic/Jórvík): eg Hall et al 2004. San Vincenzo al Volturno: eg Hodges 1997. Hamwic: eg Brisbane 1988, Morton 1992. Dorestad: eg van Es 1990.

## Appendix

Résumé

**Habitats** **fortifiés des 9^e^ et 10^e^ siècles en Europe centrale : structure, fonction et symbolisme** *par* Hajnalka Herold

La structure, les fonctions et le symbolisme des habitats fortifiés du début du Moyen-Âge (9^e^ et 10^e^ siècles) en Europe centrale, notamment dans l’Autriche, la Hongrie, la République Tchèque et la Slovaquie actuelles, sont examinés dans ce papier, qui fait un tour d’horizon de l’état actuel de la recherche. Il présente aussi de nouvelles pistes basées sur l’analyse du site de Gars-Thunau en Basse Autriche. Il met l’accent sur la position des sites fortifiés dans le paysage, les éléments de l’environnement bâti et leur organisation spatiale, ainsi que sur les tombes présentes au sein de la zone fortifiée. La région étudiée se trouvait à la frontière S.-E. de l’empire carolingien (et, plus tard, ottonien), certains des sites évoqués étant sur le territoire de l’empire de la « Grande-Moravie » des 9^e^ et 10^e^ siècles. Ces sites fournissent donc d’importants éléments de comparaison aux chercheurs travaillant dans d’autres parties de l’empire carolingien et les régions environ- nantes.

Zusammenfassung

**Befestigte Siedlungen des 9. und 10. Jh. n. Chr. in Mitteleuropa: Struktur, Funktion und Symbolik** *von* Hajnalka Herold

In diesem Artikel werden die Struktur, Funktion(en) und Symbolik frühmittelalterlicher (9.*–*10. Jh. n. Chr.) befestigter Siedlungen in Mitteleuropa, insbesondere in den heutigen Ländern Österreich, Ungarn, Tschechien und Slowakei, untersucht. Der Artikel bietet einen Überblick über den aktuellen Forschungsstand, zusammen mit neuen Erkenntnissen, die auf der Analyse der Fundstelle Gars-Thunau in Niederösterreich basieren. Besondere Betonung liegt dabei auf der Lage der befestigten Siedlungen in der Landschaft, den Elementen der bebauten Umgebung und ihrer räumlichen Organisation, sowie den Gräbern innerhalb der befestigten Bereiche. Die untersuchte Region lag an der SO-Grenze des Karolingischer- bzw. später des Ottonenreiches, und einige der besprochenen Fundstellen befanden sich im 9. und 10. Jh. n. Chr. auf dem Gebiet des ‘GGroßmährischen Reiches’. Diese Fundstellen können daher wichtige Vergleichsdaten für Forscher bieten, die sich mit anderen Teilen des Karolingerreiches und seinen Nachbargebieten beschäftigen.

Riassunto

**Stanziamenti fortificati del IX e X secolo d.C. nell’Europa centrale: struttura, funzione e simbolismo** *di* Hajnalka Herold

In questa relazione vengono esaminati la struttura, la funzione e il simbolismo degli stanziamenti fortificati altomedievali (IX*–*X sec. d.C.) nell’Europa centrale, in particolare di quelli delle odierne Austria, Ungheria, Repubblica Ceca e Slovacchia. Si tratta di una panoramica sullo stato attuale delle ricerche, e inoltre di una nuova interpretazione basata sull’analisi del sito di Gars-Thunau nella Bassa Austria. Si dà particolare rilievo alla posizione dei siti fortificati in relazione al paesaggio circostante, agli elementi che costituiscono l’ambiente edificato e la loro organizzazione spaziale, oltre che alle sepolture che si trovano all’interno dell’area fortificata. La regione oggetto di studio era situata al confine sudorientale dell’impero carolingio (e più tardi di quello ottoniano), mentre alcuni dei siti presi in considerazione, nei secoli IX e X sorgevano nel territorio della ‘Grande Moravia’. Questi siti possono quindi fornire dati comparativi importanti a quei ricercatori impegnati in altre parti dell’impero carolingio e delle regioni limitrofe.

## References

[b1] Bialeková D (1998). ‘Zur Bautechnik der Befestigungsmauer des Burgwalls in Pobedim, Bez.

[b2] Borkovský I (1953). ‘Staročeský dvorec na Levém Hradci’ [Old Bohemian court in Levý Hradec],. Archeologické Rozhledy.

[b3] Bradley R (2002). The Past in Prehistoric Societies.

[b4] Brather S (2008).

[b5] Brisbane M (1988). ‘Hamwic (Saxon Southampton): an 8th century port and production centre’, in R Hodges and B Hobley (eds), *The Rebirth of Towns in the West AD 700–1050*,. Counc Brit Archaeol Res Rep.

[b6] Brunner K (1994). 907–1156, Herzogtümer und Marken: vom Ungarnsturm bis ins 12. f ahrhundert.

[b7] von Carnap-Bornheim C, Hilberg V (2007).

[b8] Cichocki O (1999). ‘Xylotomische Untersuchungen an Holzresten aus den urnenfelderzeitlichen und frühmittelalterlichen Wallanlagen von Thunau am Kamp, MG Gars am Kamp, Niederösterreich’,. Archaeologia Austriaca.

[b9] Cs. Sós Á (1984).

[b10] Dostál B (1973).

[b11] Dostál B (1975).

[b12] Dresler P (2004). ‘Opevnění velkomoravského hradiska ve Znojmě-Hradišti a jeho vztahy k okolním lokalitám’ [The fortification of the Great Moravian hillfort in Znojmo-Hradiště and its relations to the neighbouring sites],. Sborník prací filozofické fakulty brněnské univerzity.

[b13] Dresler P (2008).

[b14] Dvorská J, Boháčová I (1999).

[b15] Dvorská J, Heußner K, Poláček L (1999).

[b16] Ettel P (2001).

[b17] Felgenhauer-Schmiedt S (1993). Das Kappele (‘die Kåpile’) ob Jadersdorf: eine spätantik-frühmittelalterliche Höhensiedlung in Oberkärnten.

[b18] Felgenhauer-Schmiedt S (2008). ‘Frühe Herrschaftsbildung im Nordwald: Die Burganlage auf der Flur Sand bei Raabs an der Thaya und die Burg Raabs’.

[b19] Fodor I (ed) 1996 The Ancient Hungarians Budapest Hungarian National Museum

[b20] Frolík J (2002).

[b21] Fusek G, Bednár P (2008).

[b22] Galuška L (2005).

[b23] Galuška L (2006). ‘Velkomoravská hradba v Uherském Hradišti – Rybárnách’ [The Great Moravian rampart at Uherské Hradiště – Rybárny],. Archeologické rozhledy.

[b24] Galuška L (2008).

[b25] Galuška L Kouřil P Měřínský Z (eds) 2001 *Velká Morava mezi východem a západem* [Great Moravia between East and West], Spisy Archeologického Ústavu AV ČR Brno **17**

[b26] Grabner M (2002). ‘Dendrochronologische Datierung der Holzfunde aus der Wehranlage Sand’,. Arbeitsberichte des Kultur und Museumsvereins Thaya.

[b27] Grabolle R (2008).

[b28] Gutjahr C (2006). ‘Der Kirchberg von Deutschfeistritz, Bezirk Graz-Umgebung, Steiermark — eine frühmittelalterliche Burgstelle?’. Arheološki vestnik.

[b29] Hahn W (1990).

[b30] Hall RA, Rollason DW, Blackburn M (2004).

[b31] Hamerow H (2006). ‘Special deposits in Anglo-Saxon settlements’,. Medieval Archaeol.

[b32] Hanuliak M, Kuzma I, Šalkovský P (1993). Mužla-Čenkov I: Osídlenie z 9.–12. storočia.

[b33] Henning J (ed) 2007 Post-Roman Towns, Trade and Settlement in Europe and Byzantium, Vol 1: The Heirs of the Roman West Berlin – New York de Gruyter

[b34] Henning J, Milo P (2007).

[b35] Henning J Ruttkay AT (eds) 1998 Frühmittelalterlicher Burgenbau in Mittelund Osteuropa Bonn Habelt

[b36] Herold H (2008). ‘Der Schanzberg von GarsThunau in Niederösterreich. Eine befestigte Höhensiedlung mit Zentralortfunktion aus dem 9.–10. Jahrhundert’,. Archäologisches Korrespondenzblatt.

[b37] Hodges R (1982). Dark Age Economics: The Origins of Towns and Trade ad 600–1000.

[b38] Hodges R (1997).

[b39] Jankuhn H (1986).

[b40] Kanelutti E (1990).

[b41] Klíma B (2001).

[b42] Költő L Vándor L (eds) 1996 *Évezredek üzenete a láp világából: Régészeti kutatások a Kis-Balaton területén 1979–1992* [The message of millennia from the world of the moor: archaeological research on the territory of the Kis-Balaton 1979–1992], Kaposvár – Zalaegerszeg: Somogy Megyei és Zala Megyei Múzeumok Igazgatósága

[b43] Kouřil P (2009).

[b44] Kovács L (1989). Münzen aus der ungarischen Landnahmezeit: archäologische Untersuchung der arabischen, byzantinischen, westeuropäischen und römischen Münzen aus dem Karpatenbecken des 10. Jahrhunderts.

[b45] Krenn M (1991).

[b46] Kučerovská T (1998).

[b47] Ladenbauer-Orel H (1996).

[b48] Macháček J (2001).

[b49] Macháček J 2007 *Pohansko bei Břeclav: ein frühmittelalterliches Zentrum als sozialwirtschaftliches System*, Studien zur Archäologie Europas **5**

[b50] Macháček J (2010).

[b51] McCormick M (2001). Origins of the European Economy: Communications and Commerce, AD 300–900.

[b52] Mitterauer M (1980). Markt und Stadt im Mittelalter, Beiträge zur historischen Zentralitätsforschung.

[b53] Morton A D (ed) 1992 *Excavations at Hamwic: Volume 1*, Counc Brit Archaeol Res Rep **84**

[b54] Müller R (1992).

[b55] Müller R (1995).

[b56] Pippal M Daim F (eds)2008 Die frühmittealterlichen Wandmalereien Mährens und der Slowakei: archäologischer Kontext und herstellungstechnische Analyse Innsbruck Wagner

[b57] Pohl W (1988). Die Awaren: ein Steppenvolk in Mitteleuropa 567–822 n. Chr..

[b58] Poláček L (2002).

[b59] Poláček L (2005).

[b60] Poláček L (2007).

[b61] Poláček L (2008a).

[b62] Poláček L (ed) 2008b *Das wirtschaftliche Hinterland der frühmittelalterlichen Zentren*, Internationale Tagungen in Mikulcice 6, Spisy Archeologického Ústavu AV ČR Brno **31**

[b63] Poláček L (2008c).

[b64] Poláček L (2009).

[b65] Poláček L Dvorská J (eds) 1999 *Probleme der mitteleuropäischen Dendrochronologie und naturwissenschaftliche Beiträge zur Talaue der March*, Internationale Tagungen in Mikulčice 5, Spisy Archeologického Ústavu AV ČR Brno **15**

[b66] Poláček L, Marek O (2005).

[b67] Polanyi K (1957).

[b68] Poleski J (2004).

[b69] Procházka R (2009).

[b70] Ruttkay AT (1998).

[b71] Ruttkay AT (2005).

[b72] Ruttkay AT (2009).

[b73] Schulze-Dörrlamm M (1993). ‘Bestattungen in den Kirchen Großmährens und Böhmens während des 9. und 10. Jahrhunderts’. Jahrbuch des Römisch-Germanischen Zentralmuseums Mainz.

[b74] Sennhauser H R (ed) 2003 Frühe Kirchen im östlichen Alpengebiet: von der Spätantike bis in ottonische Zeit München Bayerische Akademie der Wissenschaften

[b75] Šolle M (1966).

[b76] Szőke BM (1992a).

[b77] Szőke BM (1992b).

[b78] Szőke BM (2007a).

[b79] Szőke BM (2007b).

[b80] Szőke BM (2009).

[b81] Szőke BM, Éry K, Müller R (1992).

[b82] Trebsche P (2008). Die Höhensiedlung ‘Burgwiese’ in Ansfelden (Oberösterreich): Ergebnisse der Ausgrabungen von 1999 bis 2002, Linzer archäologische Forschungen **38**.

[b83] Trnka G (1981).

[b84] Tuzar J (1998).

[b85] Ulbricht I (1978).

[b86] van Es WA (1990).

[b87] von Freeden U Friesinger H Wamers E (eds) 2009 *Glaube, Kult und Herrschaft: Phänomene des Religiösen im 1. Jahrtausend n. Chr*. in Mittelund Nordeuropa, Bonn: Habelt

[b88] Westphalen P (1989).

[b89] Westphalen P (2002).

[b90] Wolf M (1996).

[b91] Wolf M (2001).

[b92] Wolfram H (1995).

